# Quality of Care in Performance-Based Financing: How It Is Incorporated in 32 Programs Across 28 Countries

**DOI:** 10.9745/GHSP-D-16-00239

**Published:** 2017-03-15

**Authors:** Jessica Gergen, Erik Josephson, Martha Coe, Samantha Ski, Supriya Madhavan, Sebastian Bauhoff

**Affiliations:** a ThinkWell, Washington, DC, USA.; b Independent consultant, Washington, DC, USA.; c University Research Co., Washington, DC, USA.; d U.S. Agency for International Development, Washington, DC, USA.; e Center for Global Development, Washington, DC, USA.

## Abstract

**Objective:**

To describe how quality of care is incorporated into performance-based financing (PBF) programs, what quality indicators are being used, and how these indicators are measured and verified.

**Methods:**

An exploratory scoping methodology was used to characterize the full range of quality components in 32 PBF programs, initiated between 2008 and 2015 in 28 low- and middle-income countries, totaling 68 quality tools and 8,490 quality indicators. The programs were identified through a review of the peer-reviewed and gray literature as well as through expert consultation with key donor representatives.

**Findings:**

Most of the PBF programs were implemented in sub-Saharan Africa and most were funded primarily by the World Bank. On average, PBF quality tools contained 125 indicators predominately assessing maternal, newborn, and child health and facility management and infrastructure. Indicators were primarily measured via checklists (78%, or 6,656 of 8,490 indicators), which largely (over 90%) measured structural aspects of quality, such as equipment, beds, and infrastructure. Of the most common indicators across checklists, 74% measured structural aspects and 24% measured processes of clinical care. The quality portion of the payment formulas were in the form of bonuses (59%), penalties (27%), or both (hybrid) (14%). The median percentage (of a performance payment) allocated to health facilities was 60%, ranging from 10% to 100%, while the median percentage allocated to health care providers was 55%, ranging from 20% to 80%. Nearly all of the programs included in the analysis (91%, n=29) verified quality scores quarterly (every 3 months), typically by regional government teams.

**Conclusion:**

PBF is a potentially appealing instrument to address shortfalls in quality of care by linking verified performance measurement with strategic incentives and could ultimately help meet policy priorities at the country and global levels, including the ambitious Sustainable Development Goals. The substantial variation and complexity in how PBF programs incorporate quality of care considerations suggests a need to further examine whether differences in design are associated with differential program impacts.

## Abstract (precis)

Structural aspects of quality such as equipment and infrastructure were the most frequently measured, with some measurement of processes of clinical care. Further examination is warranted to assess whether variations in how quality of care is incorporated into performance-based financing programs lead to differential effects.

## INTRODUCTION

Performance-based financing (PBF)—a mechanism by which health care providers or facilities earn incentives on the basis of achieving specific performance criteria—is emerging as an important tool to encourage providers and facilities to become more efficient and responsive to their clients.^1^ Because PBF allows narrow targeting of health services and requires measurement and verification of progress, it is increasingly appealing to implementers and policy makers as a path to making progress toward the health-related Sustainable Development Goals (SDGs). In recent years, PBF programs have proliferated in many low- and middle-income countries (LMICs), often with technical and financial support from donors and international agencies.^2^ For example, in 2015 the World Bank's Health Results Innovation Trust Fund supported 36 PBF programs on maternal and child health, associated with US$400 million in grants and US$2.2 billion in concessional loans.^3^

In addition to paying providers and facilities for the quantity of services provided, PBF programs also often explicitly address quality of care in their payment formulas. Quality is included either directly, by paying for specific indicators, or indirectly, by modifying the overall bonus payment according to a broader measure of quality. There are several reasons to account for quality. First, providers may compromise quality when increasing the volume of services in response to the payment incentives.^2^ Second, quality is increasingly recognized as a priority area in its own right. Third, to the extent that demand responds to quality, *increasing quality can also help achieve desired increases in service utilization*. Existing evidence indicates substantial gaps and variations in quality in many settings,^4^^–^^6^ which has contributed to the inclusion of quality in the global development agenda. For example, one of the targets for SDG 3 (ensure healthy lives and promote well-being) is to achieve … access to quality essential health care services and … quality and affordable essential medicines and vaccines ….^7^ PBF programs can potentially contribute to achieving these goals.

In addition to paying for the quantity of services provided, performance-based financing (PBF) programs also often explicitly address quality of care in their payment formulas.

However, there is little systematic evidence on the design and implementation aspects of how existing PBF programs account for quality of care. While many studies focus on an individual PBF program's impact, there appears to be substantial heterogeneity in design and operational features of such programs,^2^^,^^8^^,^^9^ reflecting the fact that PBF is comprised of a range of approaches rather than a uniform method. This variation has led to calls for better documentation of programs to better interpret impact estimates and provide practical guidance to policy makers.^8^^,^^9^

There is little systematic evidence on the design and implementation of how PBF programs account for quality of care.

In this article, we review how 32 PBF programs in 28 countries integrate quality of care within the programs' designs. Drawing on PBF program documents, we describe existing practice for how quality enters into the PBF payment formula, what quality indicators are being used, and how these measures are verified. This allows us to provide a deeper review of program parameters, describe both commonalities and variations across programs, and identify areas for further research and program development.

## METHODS

This study employed an exploratory scoping methodology to characterize the full range of quality components in PBF and potential gaps that require further research. For our purposes, we focus on the supply-side performance-based incentives that are targeted at individual health facilities, and the payments that are linked to outputs and possibly modified by quality indicators.^8^

### Identifying Programs

First, we compiled a list of known existing supply-side, health facility-based PBF programs in LMICs based on a document review of published analyses in both the peer-reviewed and gray literature. We also identified existing programs through expert consultation with a number of key donor representatives from the World Bank, Kreditanstalt Für Wiederaufbau (KfW), the U.S. Centers for Disease Control and Prevention (CDC), and the U.S. Agency for International Development (USAID). Each donor provided a list of their PBF programs and a key contact person for each, if available. We solicited programmatic information from implementers and donors primarily through email. Our research team collected and organized program manuals and accompanying tools used to measure quality performance for all facility levels (primary, secondary, and tertiary).

**FIGURE 1:**
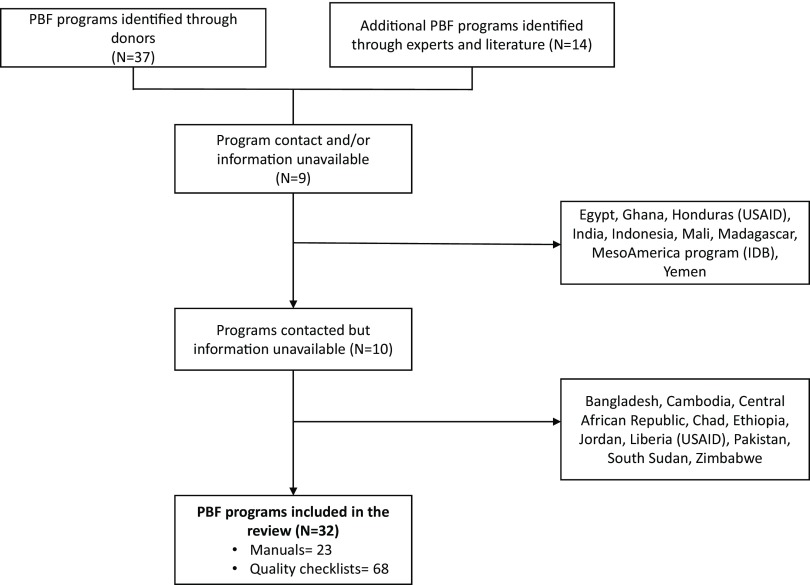
Performance-Based Financing Program Selection Process Abbreviations: IDB, Inter-American Development Bank; PBF, performance-based financing; USAID, U.S. Agency for International Development.

All programs identified were included if sufficient program information could be obtained (Figure 1). Programs were not excluded based on year of implementation, size, or phase (i.e., small-scale pilots to national implementations).

### Abstracting and Coding

We systematically reviewed each of the current (most recent) program manuals and entered information into a Microsoft Excel database that captured key quality of care program attributes, including PBF scheme (purchaser, regulator, provider), payment formula, quality assessment processes (e.g., checklists or direct observations), geographic coverage, funder, level of facility, targeted health services, and the verification process. If the information in the manual was unclear, we followed up with the implementer or donor to obtain clarification. For countries with multiple programs, we included all for which we had sufficient information.

We also collected quality checklists for all levels of care and entered the quality indicators contained in the checklists in a distinct database, including revised checklists for the same program. We copied indicators verbatim from the original checklist documents and pasted them into the database. We translated indicators in languages other than English, primarily French, and confirmed the translation with a proficient speaker. For the purposes of this study, we defined an indicator as any measure with an associated point value in a PBF quality checklist, i.e., an indicator that could affect PBF payments. Some checklists included criteria to fulfill an indicator that did not have an associated score, and these criteria were therefore not considered to be indicators. Because checklists varied in the maximum point value, we also transformed the point value into a weight that could be compared across checklists. The weight for each indicator was calculated as a percentage value of the entire checklist of its particular PBF scheme, so that the sum of all indicators' weights within an individual PBF checklist totaled to 100.

Data from the manuals and checklists were input by 3 researchers in multiple phases. Predetermined definitions were used to classify each programmatic component and indicator. After entering half of the indicators, a second researcher reviewed the database for consistency. Once all indicators were entered, the third researcher reviewed all entries. Difficult classifications were resolved through team discussions.

### Analysis

We primarily used Microsoft Excel pivot tables to compare basic characteristics across the PBF programs, including regional distribution, funding source, geographic coverage, and bonus recipient categories. Several specific analyses were conducted on the classification of payment types, verification and means of assessments, and service types.

Health facilities receiving PBF payments typically use one of two performance payment types. The first type is a "carrot-and-stick" approach that uses a combination of rewards and punishment to induce behavior change. The "carrot" refers to the quantity payment and the "stick" is a deflator associated with the quality performance, i.e., the bonus is reduced if the quality score is less than the maximum.^2^ The second type of performance payment is a carrot-and-carrot approach, consisting of a bonus payment for the quality performance that is added to the quantity payment.^2^ This dichotomy indicates whether the program rewards or penalizes a health facility based on quality performance.

PBF payments typically use one of two approaches: carrot-and-stick (rewards and punishments) or carrot-and-carrot (bonus payment on top of the quantity payment).

However, penalties and rewards can also be calculated using additional measures and in different ways. We therefore classified programs into 7 different payment types. The taxonomy for the type of payment used was developed by coding all programs on the basis of: (1) the relationships between quality and quantity, and (2) the presence of a threshold performance score. Each of the payment types are defined and visually displayed in Table 1. We retained the distinction between penalty and reward but further specified whether the payment's calculation was determined by quality performance thresholds.

**TABLE 1.**
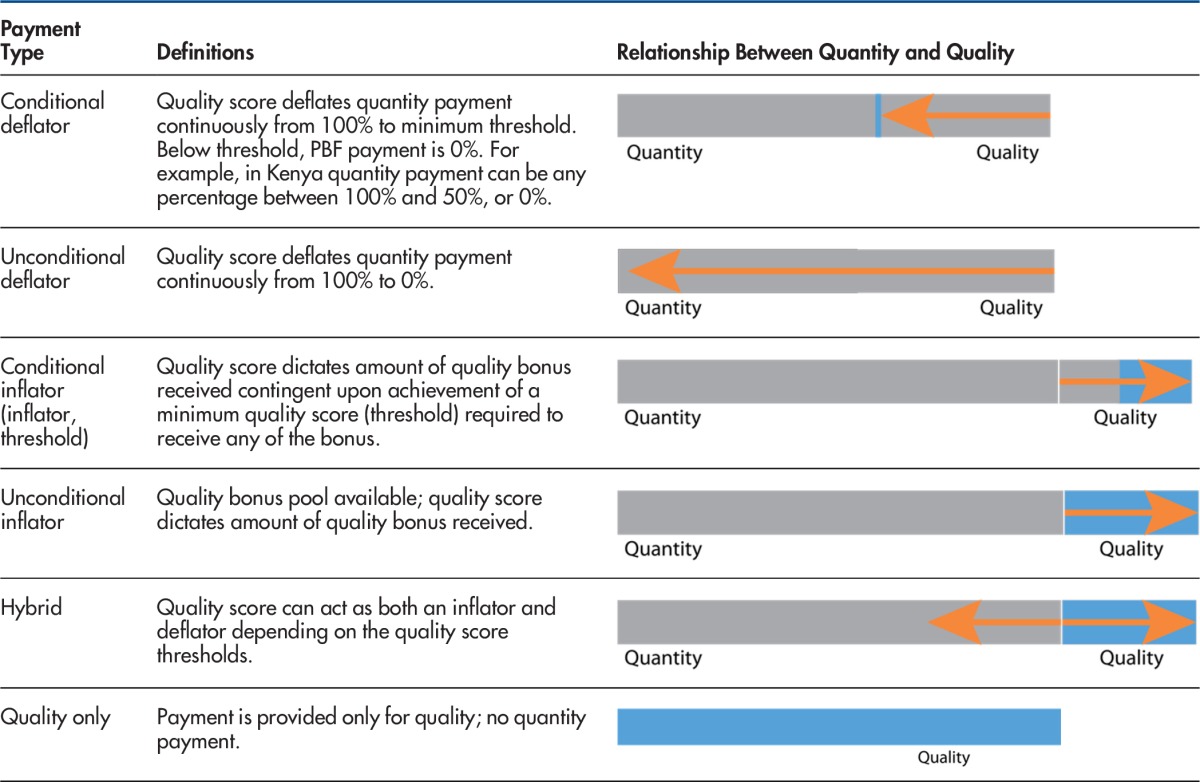
Performance-Based Financing Payment Typologies

Abbreviation: PBF, performance-based financing.

Most PBF programs purchase services conditional on the verified quality of those services. Verification is the process by which the reported quantity of services provided and the quality scores are verified externally. Many programs verify performance at multiple levels of the health system; this assessment is concerned with the health facility performance. Counter-verification, or ex-post verification, is a supplemental verification process undertaken after the PBF payment has been distributed to assess that quality services were actually received by patients, typically through patient surveys and community assessments.

Programs also vary in their means of assessment and service types. We distinguished 7 means of assessment: checklists, register review, patient record review, direct observation, staff surveys, patient surveys, and exit interviews. We aggregated health service types into 10 categories guided by the International Classification of Diseases and the International Healthcare Accreditation classifications.

### Limitations

Our analysis has several limitations. First, we obtained program information from a small set of donors. As a result, our analytic sample is skewed toward programs with involvement of these donors, and programs of a particular donor may share design commonalities across countries. Related, there is no database of PBF programs worldwide that could help us establish the relative size or representativeness of our sample. Second, we were unable to obtain complete information on all PBF programs identified, and those programs for which complete information could not be obtained were excluded from the analysis.

## RESULTS

### Analytical Sample

The final analytic sample includes 32 PBF programs initiated between 2008 and 2015 in 28 LMICs. Collectively, these interventions used 68 quality tools and 8,490 quality indicators. Comprehensive information (programmatic manual and a set of quality tools) was available for 23 PBF programs; for 9 programs we received only quality tools without manuals (Supplementary Table). Results on PBF program components are limited to those for which we received a manual. For 6 countries, we received multiple versions of revised checklists from different years. Three countries, the Democratic Republic of the Congo (DRC), Malawi, and Rwanda, had 2 concurrent PBF programs in distinct geographic regions and supported by different donors.

We assessed the quality components of 32 PBF programs, which collectively used 68 quality tools and 8,490 indicators.

### Primary Characteristics of the PBF Programs

The PBF programs included in the analysis were heavily concentrated in sub-Saharan Africa (n=21), followed by Europe and Central Asia (n=3), East Asia and the Pacific (n=2), South Asia (n=1), and Latin America and the Caribbean (n=1). The World Bank was the primary donor for 84% of the PBF programs (n=27), while a handful of programs were either partially or solely supported by other donors including USAID (n=5), the CDC (n=2), the Global Fund to Fight AIDS, Tuberculosis and Malaria (n=2), Gavi, the Vaccine Alliance (n=2), the United Nations Children's Program (UNICEF) (n=1) and KfW (n=1). A small set of the programs are cofinanced by country governments.

The PBF programs included in this review were heavily concentrated in sub-Saharan Africa, and the World Bank was the primary donor for most.

Table 2 summarizes the characteristics of the geographic coverage, funding sources, payment typologies, and incentive allocation formulas for each of the 23 programs with manuals. Program coverage was predominately subnational, with just 4 of the 23 programs achieving national coverage and significant variation in the geographic coverage for the remaining programs. For each PBF program, the incentive payments were disbursed according to allocation formulas to 3 potential facility-based recipient categories: (1) the health facility, for reinvestment in supplies, infrastructure, and related items; (2) providers, as bonuses or salary top-ups; and (3) in some cases, facility management and administrative staff, also as bonuses or salary top-ups. The median percentage allocated to health facilities was 60% and ranged from 10% in Armenia to 100% in Burundi. The median percentage allocated to health care providers was 55%, ranging from 0% in Lesotho to 80% in Burkina Faso. In Armenia, Benin, and the DRC (USAID), a portion (10% to 20%) of the total PBF payment was distributed to facility-based managerial or administrative teams. Typically, the payments were allocated to all facility-based workers or facility-based workers responsible for PBF indicators.

**TABLE 2.**
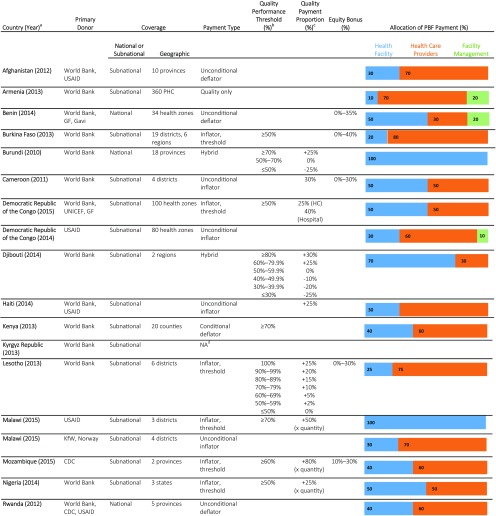

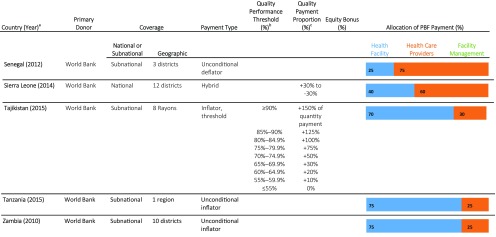
Characteristics of Quality of Care Components in PBF Programs (N= 23)

Abbreviations: CDC, U.S. Centers for Disease Control and Prevention; GF, Global Fund to Fight AIDS, Tuberculosis and Malaria; KfW, Kreditanstalt Für Wiederaufbau; PBF, performance-based financing; UNICEF, United Nations Children's Fund; PHC, primary health center; USAID, U.S. Agency of International Development.

a The table includes only the PBF programs for which we received manuals. The year denotes the version of the manual provided for this analysis.

b The Quality Performance Threshold (%) provides the performance threshold that health facilities must achieve in order to receive any quality bonus or incentive. For instance, in Burkina Faso, health facilities must score at least 50% on the quality checklist to receive a quality bonus. The amount of the bonus is quantity bonus amount ($) multiplied by quality score (50% or higher).

c The Quality Payment Proportion (%) column details the quality payment proportion if the program provides further stipulations to the quality payment calculation beyond the Quality Performance Thresholds reported in the previous column. For instance, in Djibouti, if a health facility scores above 80% on the quality checklist, it receives a quality bonus that is equal to 30% of its calculated quantity bonus. However, if a health facility scores 45% on the quality checklist, it loses 10% of its anticipated quantity bonus. The percentages provided in the Quality Payment Proportion column are the proportion of the quantity payment that is allocated due to the quality score. None of the information provided in this table differs by facility level (primary, secondary, tertiary) except for when denoted in the Quality Payment Proportion column.

d Information about the payment classification for Kyrgyz Republic was requested but the question remains unanswered.

Recipients of the PBF incentive payments included the health facility, providers, or facility management and administrative staff.

### Payment Type

In over half of the programs (n=13), performance on the quality checklists inflated the payments received by health facilities for their quantitative outputs (the carrot-carrot approach). Six of the programs were inflators without thresholds, meaning that health facilities received a quality bonus if they received a score >0%. The other 7 programs were conditional inflators with threshold scores ranging from 50% to 70% on quality checklists. Facilities had to exceed this threshold in order to increase the quantity payment.

Deflators or penalties tended to be unconditional (4 programs), meaning that the quantity payment could be deflated from 100% to 0% depending on the quality score. One program used a conditional deflator approach in which the quantity payment could deflate from 100% to the minimum threshold of 50%, and then quantity payments could be discontinuously reduced to zero if the quality score was below the threshold. Otherwise put, a minimum quality score of 50% was required to receive any PBF payment for the quarter. Three programs were hybrids, meaning that the quality score could serve as either a bonus or a penalty depending on the facility's quality score (range from +30% to −30%). The program in Armenia paid for performance solely based on quality checklists.

There were 5 programs that included what is called an "equity bonus" for certain health facilities that was calculated based on eligibility criteria ranging from 0% to 30% of the quantity payment. The allocation of the equity bonus was irrespective of the facility quantity or quality performance and was intended to ensure that incentives were sufficient for health facilities in rural or hard-to-reach areas with low population densities.

Countries with concurrent PBF programs (the DRC and Malawi) demonstrated variability in payment formulas. In the DRC, the USAID-funded project payment was based on achievement against the target, with a cap for each indicator. Quality payment was based on a quality assessment score alongside the quantity indicator score and was subject to its own target (i.e., quality score multiplied by the quality payment cap). By contrast, the program funded by the World Bank, UNICEF, and the Global Fund set the quality bonus at 25% of the quantity payment only after a facility scored 50% or above. In general, using descriptive analysis, our study did not find a relationship between payment type and related programmatic functions, for instance, allocation of incentives (between providers and facilities).

### Quality of Care Indicators

Table 3 lists the number of indicators per checklist for each of the different facility levels; only the most recent quality checklists per program are included (N=50). Across all checklists, the average number of indicators per checklist was 125 (range, 15 to 286), with an average of 146 indicators in secondary and tertiary facilities (N=19), 122 indicators for primary facilities (N=25), and 105 indicators that applied to all health facility levels (N=5). A more extensive analysis of these indicators can be found in a related paper.^10^

**TABLE 3.** Verification Process and Means of Assessing Quality of Care in PBF Programs

Country (Year)Facility Level	No. of Indicators per Checklist^a^	Verification Process		Means of Assessment^b^						
		Frequency	Verifier	Checklists No. (%)	Patient Record Review No. (%)	Register Review No. (%)	Direct Observation No. (%)	Staff Survey No. (%)	Patient Survey No. (%)	Exit Interview No. (%)
**Afghanistan (2012)**										
Secondary & Tertiary	15	Quarterly	Regional govt. team	0 (0)	1 (7)	6 (40)	0 (0)	0 (0)	8 (53)	0 (0)
**Armenia (2014)**										
Primary & Secondary	28	Biannually	Regional govt. team	0 (0)	22 (79)	6 (21)	0 (0)	0 (0)	0 (0)	0 (0)
**Benin (2014)**										
Primary	215	Quarterly	Regional govt. team	172 (80)	19 (9)	22 (10)	0 (0)	2 (1)	0 (0)	0 (0)
Tertiary	240	Quarterly	Peer-to-peer	201 (84)	22 (9)	15 (6)	1 (<1)	1 (<1)	0 (0)	0 (0)
**Burkina Faso (2011)**										
Primary	143	Quarterly	NA	64 (45)	50 (35)	29 (20)	0 (0)	0 (0)	0 (0)	0 (0)
Tertiary	119			46 (39)	53 (45)	20 (17)	0 (0)	0 (0)	0 (0)	0 (0)
**Burundi (2010)**										
Primary	188	Quarterly	Regional govt. team & NGO (patient surveys)	153 (81)	27 (14)	8 (5)	0 (0)	0 (0)	0 (0)	0 (0)
Tertiary	63	Quarterly	Peer-to-peer & NGO (patient surveys)	44 (70)	13 (21)	5 (8)	0 (0)	1 (1)	0 (0)	0 (0)
**Cameroon (2012)**										
Primary	165	Quarterly	Regional govt. team	141 (85)	14 (8)	10 (6)	0 (0)	0 (0)	0 (0)	0 (0)
Tertiary	139		Peer-to-peer	112 (81)	4 (3)	22 (16)	0 (0)	0 (0)	0 (0)	0 (0)
**Congo (2014)**										
Primary	185	NA	NA	148 (80)	6 (4)	23 (16)	7 (5)	1 (0)	0 (0)	0 (0)
Tertiary	237			185 (78)	18 (10)	25 (14)	7 (4)	2 (0)	0 (0)	0 (0)
**Democratic Republic of the Congo (2015)**										
Primary	167	Quarterly	Regional govt. team	146 (87)	10 (6)	9 (5)	1 (0)	1 (0)	0 (0)	0 (0)
Tertiary	237	Quarterly	Regional govt. team & Peer-to-peer	187 (79)	25 (11)	17 (7)	7 (3)	1 (0)	0 (0)	0 (0)
**Democratic Republic of the Congo (2012)**										
Primary	143	Quarterly	Regional govt. team & PROSANI team	130 (91)	5 (4)	6 (4)	1 (<1)	1 (<1)	0 (0)	0 (0)
Tertiary	158			137 (87)	4 (2)	17 (11)	0 (0)	0 (0)	0 (0)	0 (0)
**Djibouti (2014)**										
Primary	193	Quarterly	Regional govt. team	166 (86)	16 (8)	11 (6)	0 (0)	0 (0)	0 (0)	0 (0)
Tertiary	163		Peer-to-peer	141 (87)	12 (7)	9 (6)	0 (0)	1 (0)	0 (0)	0 (0)
**Gambia, The (2015)**										
Primary	240	NA	NA	195 (81)	6 (3)	33 (14)	0 (0)	1 (<1)	5 (2)	0 (0)
Tertiary	275			221 (80)	8 (3)	40 (15)	0 (0)	1 (<1)	5 (2)	0 (0)
**Haiti (2013)**										
Primary	147	Quarterly	NGO	131 (89)	4 (3)	11 (8)	0 (0)	1 (<1)	0 (0)	0 (0)
**Ivory Coast (2014)**										
Primary & Secondary	155	Quarterly	Regional govt. team	132 (85)	6 (4)	16 (10)	0 (0)	1 (<1)	0 (0)	0 (0)
**Kenya (2015)**										
Primary & Tertiary	85	Quarterly	Regional govt. team	78 (92)	1 (1)	6 (7)	0 (0)	0 (0)	0 (0)	0 (0)
**Kyrgyz Republic (2012)**										
Tertiary	49	Quarterly	Peer-to-peer	39 (80)	7 (14)	0 (0)	1 (2)	1 (2)	1 (2)	0 (0)
**Laos (2014)**										
Tertiary	176	NA	NA	147 (84)	25 (14)	4 (2)	0 (0)	0 (0)	0 (0)	0 (0)
**Lesotho (2014)**										
Primary	135	Quarterly	Regional govt. team	102 (76)	10 (7)	19 (14)	2 (1)	1 (<1)	1 (<1)	0 (0)
Tertiary	221			161 (73)	19 (9)	40 (18)	1 (<1)	0 (0)	0 (0)	0 (0)
**Liberia (2013)**										
Tertiary	141	NA	NA	132 (94)	1 (<1)	8 (6)	0 (0)	0 (0)	0 (0)	0 (0)
**Malawi (2015) (KfW)**										
Primary, Secondary	76	Quarterly	Regional govt. team	59 (78)	12 (16)	5 (7)	0 (0)	0 (0)	0 (0)	0 (0)
**Malawi (2015) (USAID)**										
Primary	193	Biannually	Regional govt. team	151 (78)	16 (8)	5 (3)	20 (10)	1 (<1)	0 (0)	0 (0)
**Mozambique (2012)**										
Primary & Tertiary	179	Biannually	Regional govt. team & managing NGO	97 (54)	0 (0)	4 (2)	78 (44)	0 (0)	0 (0)	0 (0)
Primary & Tertiary	81			48 (59)	29 (36)	0 (0)	3 (4)	0 (0)	0 (0)	0 (0)
Primary & Tertiary	26			15 (58)	7 (27)	4 (15)	0 (0)	0 (0)	0 (0)	0 (0)
**Nigeria (2013)**										
Primary	182	Quarterly	Regional govt. team	164 (90)	3 (2)	7 (4)	8 (4)	0 (0)	0 (0)	0 (0)
Tertiary	228	Peer-to-peer		189 (83)	21 (9)	10 (4)	7 (3)	1 (1)	0 (0)	0 (0)
**Rwanda (2012)**										
Primary	206	Quarterly	Regional govt. team & Facility management	139 (67)	10 (5)	27 (13)	30 (15)	0 (0)	0 (0)	0 (0)
**Rwanda (2009) (CHW)**										
Primary	111	Quarterly	NA	76 (68)	28 (25)	3 (3)	4 (4)	0 (0)	0 (0)	0 (0)
**Senegal (2015)**										
Primary	72	Quarterly	National & regional govt. team	61 (85)	3 (4)	7 (10)	1 (1)	0 (0)	0 (0)	0 (0)
Tertiary	109			91 (83)	6 (6)	12 (11)	0 (0)	0 (0)	0 (0)	0 (0)
**Sierra Leone (2012)**										
Primary	61	Quarterly		61 (100)	0 (0)	0 (0)	0 (0)	0 (0)	0 (0)	0 (0)
Tertiary	17		Peer-to-peer	10 (59)	1 (6)	6 (35)	0 (0)	0 (0)	0 (0)	0 (0)
**Tajikistan (2014)**										
Primary	60	Quarterly	Regional govt. team	50 (83)	6 (10)	4 (7)	0 (0)	0 (0)	0 (0)	0 (0)
Primary (Rural HC)	93			72 (77)	17 (18)	4 (4)	0 (0)	0 (0)	0 (0)	0 (0)
**Tanzania (2015) (World Bank)**										
Primary	64	Quarterly	Regional govt. team	41 (64)	6 (9)	10 (16)	0 (0)	0 (0)	7 (11)	0 (0)
Secondary	109			83 (76)	14 (13)	6 (6)	0 (0)	0 (0)	6 (6)	0 (0)
**Tanzania (2015) (Danida)**										
Primary	32	NA	NA	24 (75)	0 (0)	9 (28)	1 (3)	1 (3)	1 (3)	0 (0)
Secondary, Tertiary	44			35 (80)	4 (9)	5 (11)	0 (0)	0 (0)	0 (0)	0 (0)
**Uganda (2013)**										
Primary	26	NA	NA	8 (31)	0 (0)	8 (31)	5 (19)	0 (0)	0 (0)	5 (19)
**Vietnam (2014)**										
Primary	71	NA	NA	48 (68)	10 (14)	7 (10)	0 (0)	5 (7)	1 (1)	0 (0)
Secondary	57			27 (47)	16 (28)	10 (18)	1 (2)	2 (3)	1 (2)	0 (0)
**Zambia (2012)**										
Primary	76	Quarterly	Regional govt. team	61 (80)	3 (4)	2 (3)	10 (13)	0 (0)	0 (0)	0 (0)
Avg. No. of Indicators: 125										
Total No. of Indicators Collected: 8,490										
Avg. per Assessment Method				6,656 (78)	731 (9)	771 (9)	248 (3)	34 (<1)	45 (<1)	5 (<1)

Abbreviations: CHW, community health worker; GF, Global Fund to Fight AIDS, Tuberculosis and Malaria; govt., government; HC, health center; KfW, Kreditanstalt Für Wiederaufbau; MoPH, Ministry of Public Health; PROSANI, XXX; USAID, U.S. Agency for International Development.

Note: Quarterly equates to a 3-month period of time.

a Only the most recent quality checklist per program were included in this analysis, amounting to a total of 50 checklists.

b Definitions for Means of Assessment: checklist, a verifier physically observes and assigns a point value; direct observation of a clinical consultation by the verifier; facility register, into which detailed patient contacts with the health facility are entered; patient record, in which consultation and treatment information is recorded by providers; patient survey, assessing the quality of care through a survey of patients; staff interview obtains information and knowledge from staff; exit interview, formal meeting with patient that is leaving the facility.

On average, there were 125 indicators per checklist to assess quality of care in PBF programs.

### Health Facility Verification

Nearly all programs (91%, n=29) verified quality scores quarterly (every 3 months); the remaining 3 verified the scores biannually (Table 3). Verifiers were commonly regional government management teams, i.e., provincial, district, or health zone teams. In approximately half (8 of 15) of the programs with PBF programs at the tertiary and secondary level, the hospitals were verified using a peer-to-peer technique, in which a team of providers from one hospital verified another nearby hospital. The team composition and sampling of hospitals differed by program. All programs with complete information (n=23) included some type of counter verification, usually by an independent third party.

Nearly all PBF programs verified quality scores on a quarterly basis, usually by regional government management teams.

### Means of Assessment

The means of assessment for quality indicators varied widely among PBF programs and between health facility levels (Table 3). On average, 78% of the indicators collected were measured via checklists (6,656 of 8,490) and largely (over 90%) measured structural aspects including equipment, beds, and infrastructure. Record and register reviews each accounted for 9%, which, given the settings of these programs, required the verifier to page through multiple register books or paper-based patient records. The other assessment mechanisms included direct observation (3%) and surveys, staff interviews, and exit interviews (each <1%).

The quality indicators largely measured structural aspects such as equipment and infrastructure.

### Health Service Types

**FIGURE 2:**
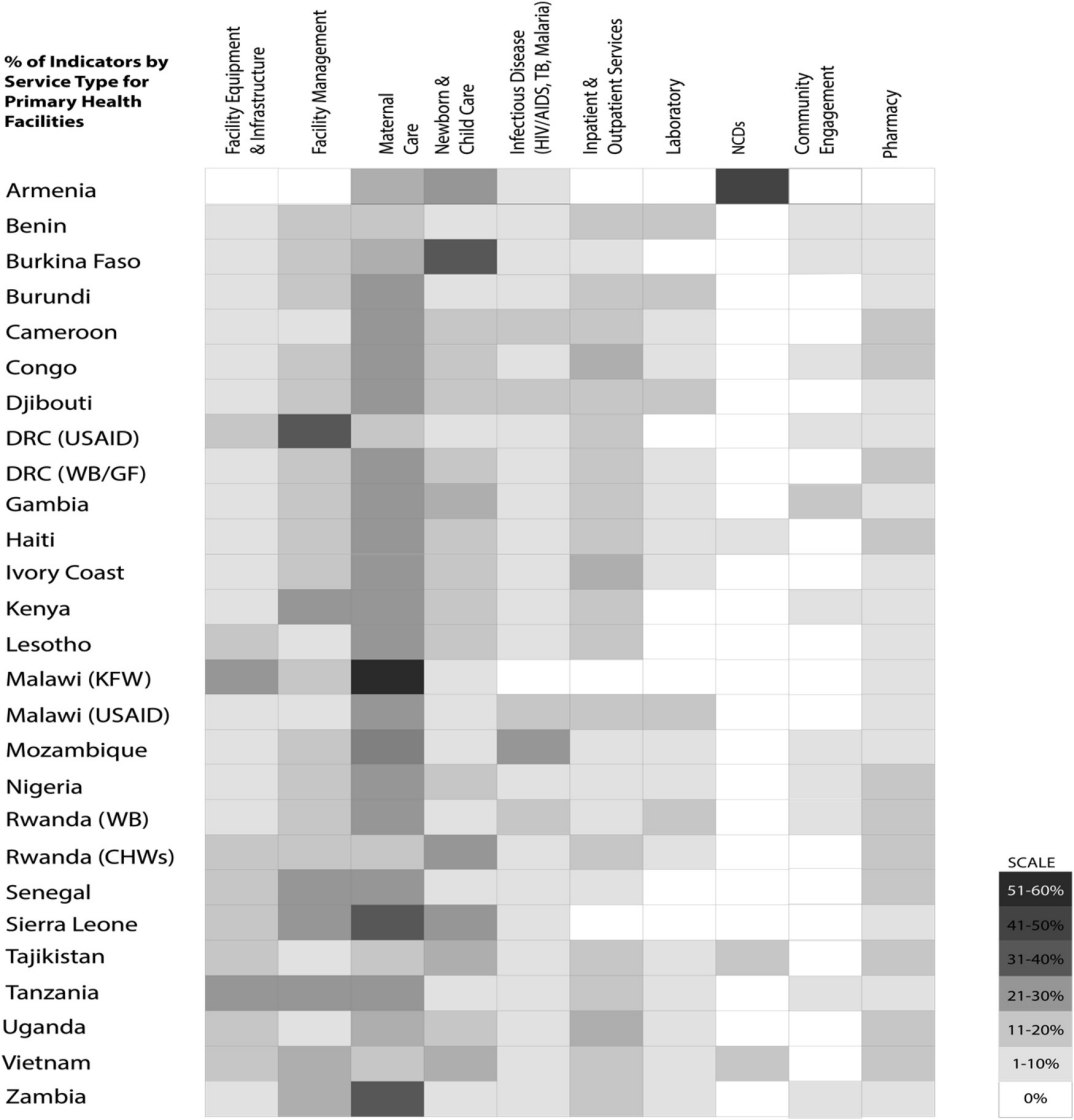
Distribution of PBF Quality Indicators by Service Type in Primary Health Facilities Abbreviations: DRC, Democratic Republic of the Congo; GF, Global Fund to Fight AIDS, Tuberculosis and Malaria; KFW, Kreditanstalt Für Wiederaufbau; NCD, non-communicable diseases; PBF, performance-based financing; TB, tuberculosis; USAID, U.S. Agency for International Development; WB, World Bank. Note: Classification of service types was guided by international standards into 10 categories to ease comparison. Inpatient and outpatient services were grouped together because the types of indicators and items being measured consisted of similar equipment and services.

**FIGURE 3:**
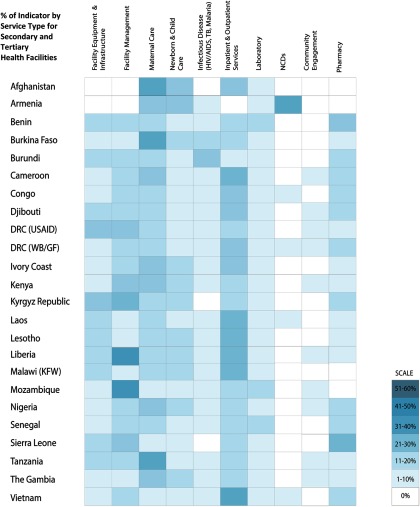
Distribution of PBF Quality Indicators by Service Type in Secondary and Tertiary Health Facilities Abbreviations: DRC, Democratic Republic of the Congo; GF, Global Fund to Fight AIDS, Tuberculosis and Malaria; KFW, Kreditanstalt Für Wiederaufbau; NCD, non-communicable diseases; PBF, performance-based financing; TB, tuberculosis; USAID, U.S. Agency for International Development; WB, World Bank. Note: Classification of service types was guided by international standards into 10 categories to ease comparison. Inpatient and outpatient services were grouped together because the types of indicators and items being measured consisted of similar equipment and services.

Figure 2 and Figure 3 show the percentage of indicators, in the most recent checklists, that measure specific types of health services; Figure 2 focuses on primary health facilities and Figure 3 on secondary and tertiary facilities. General trends were similar for primary and secondary/tertiary health facilities. Checklists emphasized maternal care and facility management, followed by newborn and child care and facility equipment.

Most of the quality checklist indicators emphasized maternal care and facility management.

On average, maternal, newborn, and child care indicators accounted for 34% of the weight of checklist points (of maximum number of points per checklist), varying between 37% for primary facilities and 28% for secondary/tertiary facilities. There was a 15% increase in the weight of points of inpatient and outpatient services from the primary facility level to the secondary/tertiary level. The increase was predominantly for structural attributes for inpatient services, such as surgical equipment and supplies.

Table 4 lists 54 of the most common PBF program indicators across 10 service delivery categories. A majority (76%) of the indicators measured structural (physical) aspects of the health facility environment, while 24% measured processes of care delivered by the health worker. Indicators categorized in facility management, infrastructure, and maternal, newborn, and child health were more common (shared) across all checklists, compared with the other service categories.

**TABLE 4.** Common PBF Quality Indicators (N=54) by Service Type^a^

Common Indicators by Service Delivery Category	No. of Checklists (% of Total Checklists)^b^
**Facility Equipment & Infrastructure**	
Presence of latrines/toilets which are sufficient and well maintained (clean, good condition, etc.)	36 (53%)
Existence of well-kept fencing around health facility buildings	30 (44%)
A communication system (radio or telephone) is effective 24/7 between health facility and next referral center(s)	21 (31%)
Existence of the health map of the geographical area is available (and displayed)	20 (29%)
Plan detailing the maintenance activities to be performed	8 (12%)
Available general inventory of all furniture and equipment	8 (12%)
Availability of electricity 24/7 (electricity, generator or solar power)	8 (12%)
**Facility Management**	
Performance or activity reports submitted on time	32 (47%)
Financial and accounting documents (including for RBF) available and well kept (bank statements, receipts, invoices etc.)	31 (46%)
Waste is treated and disposed properly in accordance with regulations of health care waste management (e.g., waste pit, placental pit, incinerator)	27 (40%)
Meeting minutes or documentation available from management or governing committee meeting	24 (35%)
HMIS data analysis report for the quarter being assessed concerning priority problems	24 (35%)
Business plan exists and is up-to-date	20 (29%)
**Maternal Care**	
All deliveries are carried out by qualified personnel	27 (40%)
Presence of proper maternity equipment (sterile clamp, maternity beds, insecticide-treated bed net)	26 (38%)
Sufficient water with antiseptic soap and liquid antiseptic in delivery room [verbatim]	24 (35%)
Weighing scale available and calibrated at zero (weight for ANC alone)	23 (34%)
Delivery room is in good condition: (1) Walls are made of solid material, are not cracked, and are plastered and painted; (2) Cement floor is not cracked; (3) Ceiling is in good condition; (4) Windows have glass and curtains; (5) Doors are in working condition; [Variable] Light 24/7, clean	22 (32%)
Book of the ANC (for mom) available – at least 10 [verbatim]	21 (31%)
Privacy (door or curtain)	22 (32%)
**Newborn & Child Care**	
Vaccination (proper administration and registry)	27 (40%)
Baby weighing and height scale available and in working condition	26 (38%)
Under-5 services (EPI, growth monitoring, curative care, health promotion) are available every day (at least 5 days a week)	22 (32%)
IMCI care protocol is applied correctly	21 (30%)
Adequate supplies for child care (1% Tetracycline eye ointment; Vitamin K)	18 (26%)
**Infectious Disease (e.g. HIV, Tuberculosis, Malaria)**	
Malaria medication in stock (Co-artemeter, Sulfadoxine/pyrimethamine, Co-trimoxazol, Quinine)	15 (22%)
Tuberculosis treatment in stock (Rifampicin, Streptomycin, Ethambutol)	14 (21%)
Correct case management of simple (uncomplicated) malaria	14 (21%)
ARI protocol correctly applied for children <5 years	13 (19%)
Well-equipped HIV counseling room ensuring privacy	13 (19%)
Correct case management of severe (complicated) malaria	12 (18%)
Knowledge of tuberculosis danger signs and criteria for referral	12 (18%)
**Laboratory**	
Available and functional microscope	23 (34%)
Availability of parasites demonstrations (GE/FS, stools, sputum) (on laminated paper, in a color book, or posters)	20 (29%)
Lab results are correctly recorded in the lab register and conform with the results in the patient booklet or lab request slip	20 (29%)
Availability of a working centrifuge	18 (26%)
Waste disposal performed correctly—organic waste in a bin with lid, safety box for sharp objects available and destroyed according to waste disposal directives	18 (26%)
**Non-Communicable Diseases (NCDs)**	
Hypertension managed according to protocol	4 (6%)
Hypertension diagnosis correctly made	2 (3%)
Counseling materials (IEC) are available for hypertension	2 (3%)
Diabetes diagnosed correctly	2 (3%)
Diabetes protocol applied	2 (3%)
Proper screening for hypertension conducted	2 (3%)
**Inpatient & Outpatient**	
Consultation room offers physical privacy	24 (35%)
Presence of a triage system with numbered cards or tokens to follow a cue	23 (34%)
Lighting available in every room (outpatient consultation and inpatient)	21 (31%)
Materials exams available in the consultation room and functional (e.g., thermometer, stethoscope, otoscope, sterile gloves, weight, tongue depressor)	21 (31%)
Examination bed available	21 (31%)
Community Engagement*	
List and mapping of community health workers	3 (4%)
**Pharmacy**	
Drugs stored properly	25 (37%)
Stock of essential drugs (paracetamol, diazepam, glucose solution, oxytocin, etc.)	18 (26%)
Pharmacy compliant with: (1) Shelves, (2) ventilated, (3) protection against direct sunlight, (4) protection against theft	17 (25%)
Stock record cards are kept accurately	17 (25%)
No expired drugs or falsified labels	15 (22%)

Abbreviations: ANC, antenatal care; ARI, acute respiratory infection; EPI, Expanded Programme on Immunization; FS, Frottis Sanguin (for blood smear) GE, Goutte Epaisse (for blood smear); HMIS, health management information system; IEC, information, education, and communication; IMCI, Integrated Management of Childhood Illness; PBF, performance-based financing; RBF, results-based financing.

a Five most “common” (frequency of indicator across entire sample of checklists) indicators are listed for each service category. In the event of a tie, we included all indicators that shared the same frequency, with the exception of community engagement (see footnote c).

b Analysis based on 68 checklists (total sample).

c Only 1 common indicator (of 68) for community engagement was observed across 3 checklists. In 2 of the checklists, there were 15+ community engagement indicators. Due to the low “commonality” of these indicators and the inability to distinguish the 5 most common indicators, we have included only the top (most frequent) indicator for community engagement.

## DISCUSSION

This study found that the quality components of PBF programs are implemented in many contexts and with high variability and complexity.^8^^,^^11^ Generally, the functional components for measuring and paying for quality (measurement tools, verification, payment formula) are consistent across programs, but the design and implementation differ.

For the programs included in this study, the quality payment formulas are split between bonuses and penalties. Within the same country (the DRC and Malawi), multiple PBF programs employ different payment formulas and allocation divisions for health care providers and facilities. Approximately half of the programs allocate 60% or more of the PBF payment (including the quality and quantity payments) to health facilities for reinvestment, while the other half allocates more than 60% to health care providers or splits it evenly between providers and facilities.

The quality payment formulas for the PBF programs included in this study are split between bonuses and penalties.

The justifications for the differences in allocation remains unexplained by donors and program designers. Moreover, it is difficult to discern what implications each payment type has on the quality of care provided and on provider behavior. PBF offers autonomy in quality investments through health facility managerial teams. However, determining the amount of a quality bonus requires knowledge of the quantity and quality score and the application of a complex formula. The implications for variable levels of fund allocation to facilities or staff and/or facility-level fiscal autonomy and strategic investment on quality dimensions, such as infrastructure and equipment, clinician competency, and patient satisfaction efforts, remains understudied.

It is difficult to discern what implications different payment types have on the quality of care provided and on provider behavior.

Notably, the verification process is the most consistent across regions, with similar teams carrying out the verification on quarterly schedules. This is likely due to the availability of regional management teams already on the government payroll or the availability of donor-supported NGOs contracted to undertake the verification. This heavy reliance on regional management teams points to a common challenge faced by many countries—that is, the additional burden placed on these District Health Management Teams or equivalent regional teams to undertake regular verification of PBF facilities on an average of 125 indicators per facility. In addition, deploying district officials to conduct verifications could generate a conflict with their other roles, for example, to constructively monitor and support providers.^12^ In the case of hospitals, peers may not be effective at verification—but in some contexts they may be some of the few experts qualified to assess quality. These issues point to the logistical and operational constraints in which PBF programs operate and also affirm the need for a well-articulated theory (or set of theories) of change for PBF.^13^

Moreover, results from Table 3 point to a reliance on the checklist for assessment, limiting the utility of the PBF program to effect improvements in certain aspects of quality. Mixed modalities of assessment can address quality of care more holistically. For example, exit interviews and direct observation can inform the experience and provision of care while provider interviews can shed light on motivation. (Additional detail about indicator typology and measurement can be found in a related paper.^10^) Selection of assessment methods is likely informed by trade-offs between cost and quality of data. For instance, register and patient record reviews may be less costly, but the quality data may vary. In Rwanda, patient record review, verified by qualified supervisors, were considered a valuable quality criterion, resulting in systemic improvements in data collection, monitoring, and supervision that contributed far more to the quality improvements than service delivery improvements.^14^ Direct observations may yield good quality at relatively higher cost. One potential solution is to always conduct register reviews and supplement with direct observations for a random sample of facilities, hence maintaining this thorough measurement but at a lower overall cost. Moreover, the findings from Table 3 suggest that more cost-effective methods of assessment may need to be developed and/or employed such as clinical vignettes and tablet- or smartphone-based verification. Indeed, cost-effectiveness itself of different verification methods should be assessed to inform the selection of one method over another or a justification for using mixed methods.

Using mixed methods to assess quality in PBF programs could address quality of care more holistically but most programs relied on checklists.

There is also consistency in quality assessment of service types. Figure 2 and Figure 3 demonstrate a clear preference for incentivizing maternal, newborn, and child health services and inpatient and outpatient services, suggesting a focus on burden of disease (mortality and morbidity). This could reflect homogeneity in policy priorities of the countries or donors, including the maternal, newborn, and child health focus of the Health Results Innovation Trust Fund of the World Bank, involved in these programs. Community engagement, non-communicable diseases, and pharmacy appear to have the fewest associated indicators, suggesting that these may be hardest to measure (community engagement), represent relatively low burden of disease or surveillance in the included countries (non-communicable diseases), or hardest to effect systemic improvements (supply chain in pharmacy) using PBF.

Our study also highlights the need for more systematic documentation. Theoretically, PBF should offer a wealth of data on quality of care given the length and frequency of measurement; however, this information remains hard to access by all actors. For policy makers and PBF practitioners, there is no comprehensive central repository for PBF program manuals and quality tools. The current structure of PBF manuals and quality checklists, long documents in PDF format, is not conducive to information sharing and aggregation, so the current state of practice has been unknown up to this point. Performance data from quality tools is inaccessible on a country or health facility level, with the notable exception of the PBF portal, which is an online platform that displays quantity and quality data at the facility level for select countries.^15^ Although the portal is an important first step, sharing of health facility performance per quality indicator is required to better understand what types of quality measures are well suited for PBF. The growing PBF Community of Practice could be a good place to house both programmatic documentation and available performance data.^16^

While our findings shed light on the current and past state-of-practice of addressing quality in PBF, they raise further questions. The observed differences in payment formula and allocation, service types, and length of the tools call for further examination of why each program is unique and the justification for the differences, and most importantly whether differences in design are associated with differential program impacts. Future foundational research could model the various incentives we identified in real-life PBF programs, also to characterize which approaches may be most effective, at least in theory. Specific research gaps related to program operations include detailed performance data and the percentage of incentives paid based on quality, leading to the cost-benefit to management and providers for completing the quality tool and investing in quality improvement measures. There is also the black box of PBF costs; calculating time costs to facility staff and quality-specific costs, predominantly verification costs. These costs and benefits should be compared with those of other quality assessment methods that are already being used like supportive supervision, accreditation, and independent quality evaluations by NGOs.

Future research could model the various incentives that we've identified in the PBF programs included in this study to characterize which approaches may be most effective.

## CONCLUSIONS

PBF is a potentially appealing instrument to address shortfalls in quality of care and, ultimately, to help meet policy priorities at the country and global levels, including the ambitious goals set forth in the SDGs. As our review of 32 PBF programs highlights, there is substantial variation and complexity in how programs incorporate quality of care considerations. There are differences in how quality is incorporated in the payment formula, how many and what indicators are included in checklists, and how they are measured. While PBF programs should be aligned with local conditions and they need to primarily focus on executing payments, the heterogeneity and similarities between programs suggests scope for learning how these programs can more effectively incentivize and support providers to address gaps in quality.^11^ More research and policy effort is urgently needed to make the best use of PBF as a targeted supply-side intervention.

PBF is a potentially appealing instrument to address shortfalls in quality of care.

## Supplementary Material

Supplemental material

## Notes

**Peer Reviewed**

**First Published Online:** 2017 Mar 15
