# Supplementary material for: Quality of Care in Performance-Based Financing: How It Is Incorporated in 32 Programs Across 28 Countries
Source: Glob Health Sci Pract. 2017 Mar 15;5(1):90–107. doi: 10.9745/GHSP-D-16-00239 (PMC5493453; doi:10.9745/GHSP-D-16-00239)
Supplement: Supplemental material [file supp_5_1_90__index.html]

Quality of Care in Performance-Based Financing: How It Is Incorporated in 32 Programs Across 28 Countries — Supplemental material 

# Quality of Care in Performance-Based Financing: How It Is Incorporated in 32 Programs Across 28 Countries

## Supplemental material

- Text s01, PDF - Text s01, PDF
