# Supplementary material for: Quality of Care in Performance-Based Financing: How It Is Incorporated in 32 Programs Across 28 Countries
Source: Glob Health Sci Pract. 2017 Mar 15;5(1):90–107. doi: 10.9745/GHSP-D-16-00239 (PMC5493453; doi:10.9745/GHSP-D-16-00239)
Supplement: Supplemental material [file GHSP-D-16-00239_16-00239-Gergen-Supplementary_material.pdf]

Gergen J, Josephson E, Coe M, Ski S, Madhavan S, Bauhoff S. Quality of care in performance-based financing: how it is incorporated in 32 programs across 28 countries. Glob Health Sci Pract. 2017;5(1). http://dx.doi.org/10.9745/GHSP-D-16-00329

Supplement Table. Sample of Performance-Based Financing (PBF) Programs (N=32) and Quality Checklists (N=68) Included in the Review

		Materials Included in This Assessment			
Country (Year)	Primary Donor	Manual	Checklist	No. of Checklists	Health Level
Afghanistan (2012)	World Bank, USAID	X	X	1	Secondary/Tertiary (2012)
Armenia (2013)	World Bank	X	X	1	Primary/Secondary (2014)
Benin (2014)	World Bank, GF, Gavi	X	X	4	Primary (2010)<br>Tertiary (2010)<br>Primary (2014)<br>Tertiary (2014)
Burkina Faso (2013)	World Bank		X	2	Primary (2011)<br>Tertiary (2011)
Burundi (2010)	World Bank	X	X	2	Primary (2010)<br>Tertiary (2010)
Cameroon (2011)	World Bank	X	X	2	Primary (2012)<br>Tertiary (2012)
Congo – Brazzaville (2013)	World Bank		X	2	Primary (2014)<br>Tertiary (2014)
Democratic Republic of the Congo (2015)	World Bank, UNICEF, GF	X	X	2	Primary (2015)<br>Tertiary (2015)
Democratic Republic of the Congo (2014)	USAID	X	X	2	Primary (2011)<br>Tertiary (2012)
Djibouti (2014)	World Bank	X	X	2	Primary (2014)<br>Tertiary (2014)
Gambia, The (2014)	World Bank, HRITF		X	2	Primary (2015)<br>Tertiary (2015)
Haiti (2014)	World Bank, USAID	X	X	1	Primary (2013)
Ivory Coast (2014)	World Bank	X	X	1	Primary/Secondary/Tertiary (2014)
Kenya (2013)	World Bank	X	X	3	Primary/Tertiary (2012)<br>Primary (2013)<br>Primary/Tertiary (2015)
Kyrgyz Republic (2013)	World Bank		X	1	Tertiary (2012)
Laos (2014)	World Bank		X	1	Tertiary (2014)

Gergen J, Josephson E, Coe M, Ski S, Madhavan S, Bauhoff S. Quality of care in performance-based financing: how it is incorporated in 32 programs across 28 countries. Glob Health Sci Pract. 2017;5(1). http://dx.doi.org/10.9745/GHSP-D-16-00329

				Materials Included in This Assessment	
**Lesotho (2013)**	World Bank	X	X	4	Primary (2013)<br>Secondary (2013)<br>Primary (2014)<br>Secondary (2014)
**Liberia (2013)**	World Bank		X	1	Tertiary (2013)
**Malawi (2015)**	USAID	X	X	1	Primary (2015)
**Malawi (2015)**	KfW, Norway	X	X	2	Primary/Secondary/Tertiary (2014)<br>CEmONC (2015)
**Mozambique (2015)**	CDC	X	X	3	Primary, IMM (2012)<br>Primary, IMQ (2012)<br>Primary/Secondary/Tertiary, PCI (2011)
**Nigeria (2014)**	World Bank	X	X	4	Primary (2011) Tertiary (2011) Primary (2013) Tertiary (2013)
**Rwanda (2012)**	World Bank, CDC, USAID	X	X	3	Primary (2008)<br>Primary (2012)<br>CHWs (2009)
**Senegal (2012)**	World Bank	X	X	6	Primary (2012)<br>Secondary (2012)<br>Primary (2013)<br>Secondary (2013)<br>Primary (2015)<br>Secondary (2015)
**Sierra Leone (2014)**	World Bank	X	X	3	Tertiary (2012)<br>Primary (2012)<br>Cross-cutting (2012)
**Tajikistan (2015)**	World Bank	X	X	2	Health House (2014)<br>Rural Health Center (2014)
**Tanzania (2015)**	World Bank	X	X	2	Primary (2015)<br>Secondary (2015)
**Tanzania (2015)**	Danida		X	3	IPC (2013)<br>Primary (2015)<br>Secondary/Tertiary (2015)
**Uganda (2011)**	UK Aid		X	1	Primary (2013)
**Vietnam (2013)**	World Bank		X	2	Primary (2014)<br>Secondary (2014)
**Zambia (2010)**	World Bank	X	X	2	Primary (2010)<br>Primary (2012)

Abbreviations: CDC, U.S. Centers for Disease Control and Prevention; CEmONC, comprehensive emergency obstetric and newborn care; CHW, community health worker; GF, Global Fund to Fight AIDS, Tuberculosis and Malaria; HRITF, Health Results Innovation Trust Fund; IMM, Instrumento da Melhora da Qualidade; IMQ, Iniciativa Maternidade Modelo; PCI, Prevention and Control of Infections ; KfW: Kreditanstalt Für Wiederaufbau; USAID, U.S. Agency of International Development.
